# Alloantibody Generation and Effector Function Following Sensitization to Human Leukocyte Antigen

**DOI:** 10.3389/fimmu.2016.00030

**Published:** 2016-02-04

**Authors:** Michelle J. Hickey, Nicole M. Valenzuela, Elaine F. Reed

**Affiliations:** ^1^Department of Pathology and Laboratory Medicine, UCLA Immunogenetics Center, University of California Los Angeles, Los Angeles, CA, USA

**Keywords:** human leukocyte antigen, allorecognition, HLA antibody, non-HLA antibody, transplant, Fc receptor, complement, endothelium

## Abstract

Allorecognition is the activation of the adaptive immune system to foreign human leukocyte antigen (HLA) resulting in the generation of alloantibodies. Due to a high polymorphism, foreign HLA is recognized by the immune system following transplant, transfusion, or pregnancy resulting in the formation of the germinal center and the generation of long-lived alloantibody-producing memory B cells. Alloantibodies recognize antigenic epitopes displayed by the HLA molecule on the transplanted allograft and contribute to graft damage through multiple mechanisms, including (1) activation of the complement cascade resulting in the formation of the MAC complex and inflammatory anaphylatoxins, (2) transduction of intracellular signals leading to cytoskeletal rearrangement, growth, and proliferation of graft vasculature, and (3) immune cell infiltration into the allograft via FcγR interactions with the FC portion of the antibody. This review focuses on the generation of HLA alloantibody, routes of sensitization, alloantibody specificity, and mechanisms of antibody-mediated graft damage.

## Introduction

The immune response is designed to recognize antigens that are distinct from self – termed “non-self” or “altered self” – be they protein, lipid, or carbohydrate. Allorecognition is the activation of the transplant recipient’s adaptive immune response to foreign histocompatibility antigens following transplant (1, 2). This review focuses on the recognition of allogeneic human leukocyte antigen (HLA) and non-HLA molecules by the humoral immune response in the context of transplantation. We discuss the generation of alloantibodies, and how they mediate graft injury and rejection.

### Human Leukocyte Antigen: Genomic Organization, Structure, Polymorphism, and Function

The human major histocompatibility complex (MHC), located on chromosome 6, is composed of highly polymorphic HLA class I genes (HLA-A, -B, and -C), HLA class II genes (HLA-DR, -DQ, and -DP), non-classical class I genes (HLA-E, -F, and -G), and class I-like genes (MICA and MICB) (3). The HLA class I molecules function to present peptide derived from intracellular antigens to CD8+ T lymphocytes and serve as ligands for receptors on natural killer (NK) cells. The HLA class II molecules present antigens from the extracellular space to CD4+ T cells.

**Figure 1:**
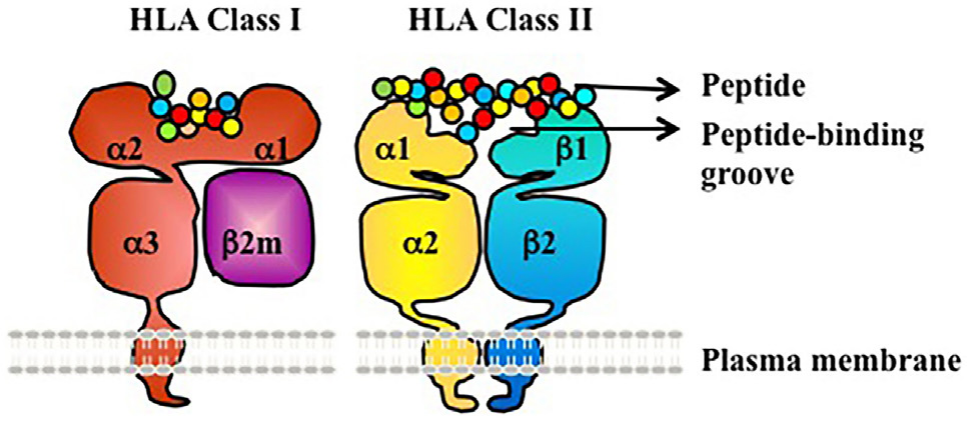
**HLA class I and II are heterodimeric transmembrane proteins**. HLA Class I is made up of a heavy chain with three globular domains (a1, a2, and a3) non-covalently bound to β2m. HLA Class II is made up of two heavy chains (a-chain and b-chain) each with two globular domains (a1 and a2 or b1 and b2). The a1and a2 domains of HLA class I, and the a1 and b1 domains of HLA class II, make up the peptide-binding groove.

Human leukocyte antigen molecules are heterodimers formed by polypeptides encoded by two distinct genetic loci (Figure 1) (3). The HLA class I molecule consists of one heavy β-chain that is non-covalently bound to a β_2_-microglobulin (β_2_m) light chain at the cell surface for stability. β2m is highly conserved and does not exhibit polymorphism. The HLA class II molecule is composed of two transmembrane glycoprotein chains – an α-chain and β-chain. The α-chain shared by all HLA-DR molecules (DRA1) has limited polymorphism (seven alleles identified to date, with only two different proteins/amino acid sequences; the amino acid polymorphism is V217L in the cytoplasmic domain) and is not a known target of humoral alloresponses. By contrast, both the α- and β-chains of HLA-DP and HLA-DQ are polymorphic (3, 4).

Globular domains of the HLA Class I and II molecules form the peptide-binding cleft that accommodates peptide antigens and interacts with the T cell receptor (TCR). The remarkable polymorphism of HLA Class I and II molecules allows for the presentation of a vast array of antigenic peptides within the human population. Each HLA molecule binds distinct peptides. At the protein level, HLA molecules are defined as antigens by either low-resolution (two digit, serologic level) or high resolution (four digit, allele level) nomenclature (Table 1). At the serologic level, there are about 20 HLA-A, 50 HLA-B, 10 HLA-Cw, 18 HLA-DR, and 7 HLA-DQ antigens. However, at the allele level of resolution, the number of HLA antigens in each serogroup is tremendously expanded due to genetic polymorphism within each serogroup – ~2000–3000 distinct proteins for each of HLA-A, B, and C, ~500–2000 for each of DRB1, DQB1, DPB1, and ~10–50 for each of DRB3 (DR52), DRB4 (DR53), DRB5 (DR51), and DQA1(4). Amino acid differences between HLA alleles enable presentation of a diverse array of peptides, and represent the basis for alloimmune recognition of non-self HLA by both T cells and antibodies (3).

**Table 1:** **Typing of HLA molecules can be at low or high resolution**.

Level of typing resolution	Definition	Nomenclature
Low	Serologic/antigen	A2
High	Allele	A*02:01

### Mechanisms of Allorecognition and Generation of Allospecific Antibodies

**Figure 2:**
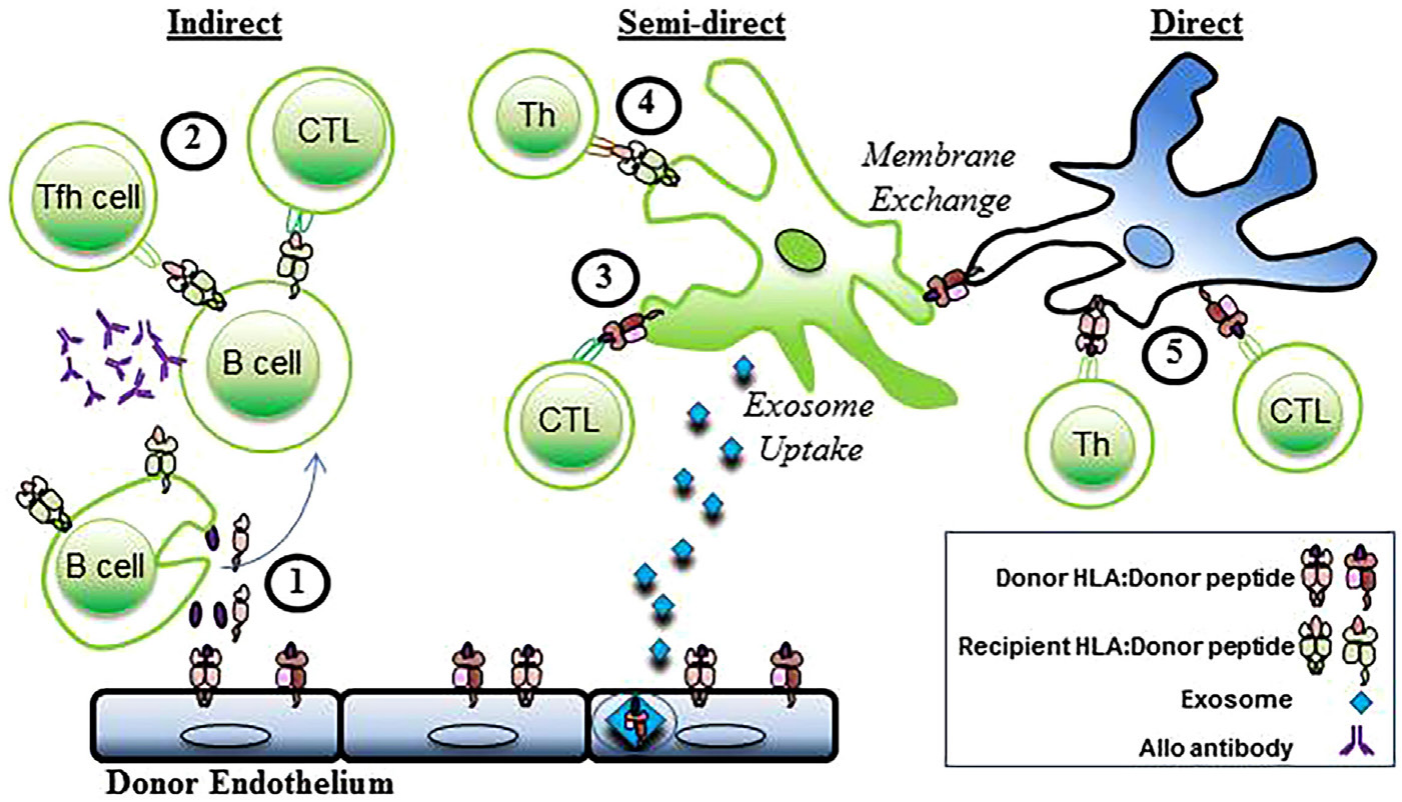
**Indirect, semidirect, and direct allorecognition**. In the indirect pathway, (1) donor alloantigens are processed by recipient B cells and (2) presented to recipient T follicular helper (T_FH_) cells and CTL. Alloantibodies are generated when alloreactive B cells interact with CD4+ T cells. The semidirect pathway involves (3) intact donor HLA class I:peptide complexes that are presented on the DC of the recipient (through either membrane exchange or exosome uptake) to recipient CD8+ T cells (CTL). Simultaneously, (4) processed donor peptide is presented in the context of the recipient’s HLA class II to the recipient’s helper CD4+ T cells (Th). In the direct pathway, (5) allogeneic MHC class I and II antigens are presented to recipient CD4+ and cytotoxic CD8+ T cells (CTL) by donor APCs. Recipient cells, green. Donor Cells, blue.

Three distinct pathways of allorecognition have been defined (Figure 2). The direct, indirect, and semidirect pathways can occur independently or simultaneously. Activation of the recipient’s CD4+ T lymphocytes is a pivotal step in the initiation of the immune response to alloantigen following transplantation leading to downstream activation of cytotoxic CD8+ T lymphocytes and antibody-producing B cells.

#### Indirect Allorecognition

Indirect allorecognition is the activation of the transplant recipient’s CD4+ T cells by alloantigen that is processed and presented in the context of the recipients HLA as occurs with the normal immune response to foreign pathogen (2). Donor antigens, shed by the grafted organ, are processed and presented in the context of self-restricted HLA class II by the recipient’s B cells. The recipient’s follicular helper CD4+ T cells are then activated to provide help leading to the generation of alloreactive CD8+ effector T cells and antibody-producing B cells (1, 5, 6). The immune response engendered by this pathway is credited with driving chronic rejection and due to lower frequency of T cells with indirect allospecificity, and requirement for antigen processing, is physio-dynamically slower than the response to presentation through the direct pathway (7–10).

#### Direct Allorecognition

Direct allorecognition is the activation of the transplant recipient’s CD4+ T cells by donor HLA:peptide complexes (2). Antigen presentation is mediated by the donor’s dendritic cells that are transplanted as passengers with the organ. In the context of inflammatory signals subsequent to the transplantation surgery, the donor’s DC, presenting intact donor allo-histocompatibility antigens, migrate to the secondary lymph nodes of the recipient and present antigen to the recipients CD4+ T cells (11, 12). The strength of the immune response elicited by the direct allorecognition pathway correlates to the high frequency of recipient allogeneic T cells that become activated during the first few weeks following transplant (13, 14) mediating acute rejection. The immune response weakens as the passenger DC leave the graft (15, 16). CD4+ T cells activated through the direct pathway are capable of providing help to effector CD8+ T cells, therefore, promoting rejection of the transplanted organ (5). However, activation of B cells and production of alloantibody does not occur in the context of direct allorecognition as there is no cognate interaction between the T helper cell and B cell (5).

#### Semi-Direct Allorecognition

The semi-direct pathway of allorecognition is presented as a hypothesis to describe events of apparent overlap between the direct and indirect pathways. Evidence from animal models of transplant rejection indicate that indirect allospecific CD4+ T cells can provide help to direct allospecific CD8+ T cells (17, 18). In principle, this would require a “four cell” model in which CD4+ T cells activated via the indirect pathway by processed alloantigen in the context of self-restricted HLA class II provide help to effector CD8+ T cells activated via the direct pathway by donor passenger APC bearing intact HLA:peptide. The “four cell model” challenges the dogma of the “three cell” or “linked” model whereby the primary mechanism by which activated helper T cells provide help to effector CD8+ T cells is by providing signals to the APC that result in the upregulation of presented antigens (19–21). Helper CD4+ T cells, therefore, “license” APC to more effectively present peptide in the context of HLA class I. The “three cell” model requires that both antigenic determinants recognized by CD4+ and CD8+ T cells be presented on the same APC.

However, the mechanism underling the phenomena of semi-direct allorecognition more likely lies in the exchange of membrane proteins between immune cells (22). After transplantation, the recipients DC acquire intact donor HLA class I:peptide complexes from donor passenger DC or endothelial cells through either cell–cell interactions or by uptake of exosomes containing the antigen that are shed from donor tissue (23, 24). In following, the recipients DC now bears intact donor HLA class I molecules as well as recipient HLA class II molecules, and is capable of stimulating the recipients CD4+ and CD8+ T cells via the indirect and direct pathways in a “three cell” model. Soluble MHC class I can be taken up by DC *in vitro*, and then presented leading to the production of alloantibody (25). The work by Curry et al. implies that soluble alloantigen can be taken up and presented intact to direct B cells, and can simultaneously be processed and presented to indirect CD4+ T cells.

### Generation of HLA Alloantibody

Conlon et al. (6) definitively showed that production of alloantibody occurs exclusively through the indirect pathway. In a murine heart allograft model, C57B/6 mice (H-2^b^) lacking intact TCRs were transplanted with a BALB/c allograft (H-2K^d^). Subsequent reconstitution with TCR transgenic CD4+ T cells engineered to specifically recognize an immunodominant BALB/c peptide (H-2K^d^_54–68_) processed and presented by MHC Class II resulted in a strong anti-H-2K^d^ IgG alloantibody response to the allograft. Furthermore, the adoptively transferred CD4+ T cells were found in germinal centers (GC), having acquired the phenotype of T follicular helper (T_FH_) cells (CXCR5^+^CCR7^−^), and anti-H-2K^d^ plasma cells were found in the bone marrow. By contrast, direct-pathway CD4+ T cells were unable to provide help to allospecific B cells and alloantibody was not produced.

#### Formation of the Germinal Center and Generation of Long-Lived Memory

B cells residing in the secondary lymphoid organs can be exposed to small antigens directly through diffusion from the lymphatic system, or to large immune complexed antigens presented by follicular dendritic cells or by macrophages. Regulation of B cell immunity and generation of antibody-secreting plasma cells is primarily dependent on interactions with T_FH_ cells in the GC of the secondary lymphoid organs (26). Antigen-specific T_FH_ cells and antigen-primed B cells migrate to follicular regions of the secondary lymph nodes and form stable contacts through the signal lymphocyte activation molecule (SLAM)-associated protein (SAP) (27). Integrins and the SLAM protein CD84 are also involved in the interaction between T_FH_ cells and pre-GC B cells (28). These interactions ultimately lead to significant proliferation of antigen-specific B cells and the formation of the GC. CD4+ T cells recognize their cognate peptide antigen presented in HLA class II by the B cell, and provide help through costimulation and cytokines to drive activation and clonal expansion of B cells.

In the GC, B cells make contact with T_FH_ cells that are both transient and stable resulting in selection of B cells that will that enter the long-lived memory component of the immune system (29, 30). Here, through somatic hypermutation, GC B cells that have high antigen affinity differentiate into memory B cells or antibody-producing long-lived plasma cells (31). Activated B cells differentiate into low-affinity antibody-producing plasmablasts, or undergo class-switch recombination and somatic hypermutation to form affinity matured, class-switched memory B cells or plasma cells. Long-lived plasma cells residing in the bone marrow contribute to much of the circulating antigen-specific immunoglobulin and can persist for decades. There is also evidence that memory B cells can be maintained in the circulation without a requirement for continuous antigen exposure (32), ready for rapid recall upon repeated stimulation with antigen.

Recent work has aimed at detection of circulating allospecific memory B cells to predict durable sensitization and anamnestic responses in patients awaiting transplantation. One recent report (33) found that circulating HLA-specific B cells were found only in patients with a history of sensitization, and were detectable in nearly half of such patients. Interestingly, patients with circulating HLA antibodies but no known sensitization event had no detectable circulating B cells. Transfusion also resulted in little to no detectable circulating anti-HLA memory B cells, consistent with the theory that transfusion is a less vigorous sensitizing event compared with pregnancy or transplantation (see below) (34). Snanoudj et al. were able to detect circulating B cells targeting prior donor antigens many decades after transplantation and even after graft removal (33), supporting the paradigm that memory B cells do not require persistent antigen for survival. Finally, and most notably, several patients had detectable HLA antibody secreting B cells in circulation but no detectable circulating antibodies in their sera.

### Kinetics of Allorecognition

Direct pathway-activated donor-specific T cells are associated with acute T cell-mediated rejection in renal transplant patients (35). CD4+ T cells isolated from the recipient’s pre-transplant blood that were responsive to direct allostimulation with donor cells were also found to be predictive of early post-transplant outcomes (35, 36). However, T cells activated via the direct pathway were found to be predominantly hyporesponsive in patients with transplant coronary artery disease (TCAD), chronic allograft nephropathy (CAN), or chronic rejection following liver transplant indicating that these cells are not contributing to chronic rejection (7, 10, 37). In comparison, T cells primed by the indirect pathway are thought to mediate chronic rejection and are found in high frequency in patients with CAN, and in heart transplant patients with chronic rejection (7, 9, 10, 38).

Notably, T cells stimulated by the indirect allorecognition pathways are also capable of contributing to acute rejection during the early post-transplant period. Circulating allopeptide-reactive T cells were predictive of rejection in heart transplant patients studied during the first 10 weeks post-transplant (39). Furthermore, T cells responsive to allopeptide were found in significant quantities above that found in circulation when isolated from biopsies of graft tissue, suggesting that indirect pathway T cells can contribute directly to acute graft rejection (39).

## Alloantibody Antigen Specificity

### Antibody Structure and Function

Antibodies are heterodimers composed of a light chain and heavy chain encoded by distinct loci on different chromosomes. Each chain contains a constant region that is invariant, and a variable region that undergoes both recombination and somatic hypermutation to yield clonally unique sequences. The variable regions of both heavy and light chain form the antigen binding region (“complementarity determining region”), or paratope, which binds its cognate epitope on the antigen. Human immunoglobulins are divided into five isotypes (IgM, IgD, IgA, IgE, and IgG). Several of these isotypes are further divided into subclasses (IgG1, IgG2, IgG3, and IgG4; IgA1 and IgA2). Antibody isotype and subclass are determined by the constant region.

The subclasses were identified and numbered according to their predominance in circulation rather than order on the genome. Early in the GC reaction, IgM+ B cells class switch first to IgG3 or IgG1, then IgG2, and rarely IgG4 [immunoglobulin sequential class switching is described in Ref. (40, 41)].

Functionally, the subclasses of IgG are distinct. IgG1 has the highest concentration in circulation, and fixes complement well. IgG2 is the next most abundant in circulation and is not an efficient complement fixer. IgG3 is unique with its long hinge region that confers the highest affinity for C1q compared with other subclasses, making it a potent effector [extensively reviewed in Ref. (42)]. However, IgG3 has the shortest half-life in circulation and, being first in order of class switching, has typically the lowest affinity for antigen but is the most potent activator of complement (43). IgG1, IgG3, and IgG4 mostly recognize protein antigens, while IgG2 is canonically efficient at recognizing carbohydrate antigens (in the absence of T cell help) and allergens. It is thought that IgG2 and IgG4 appear later after class switching and affinity maturation, as they have higher affinity for antigen but generally less effective activation of Fc-mediated effector functions, to temporally limit the immune response (41).

### Antibodies Are Specific for Antigenic Epitopes

**Figure 3:**
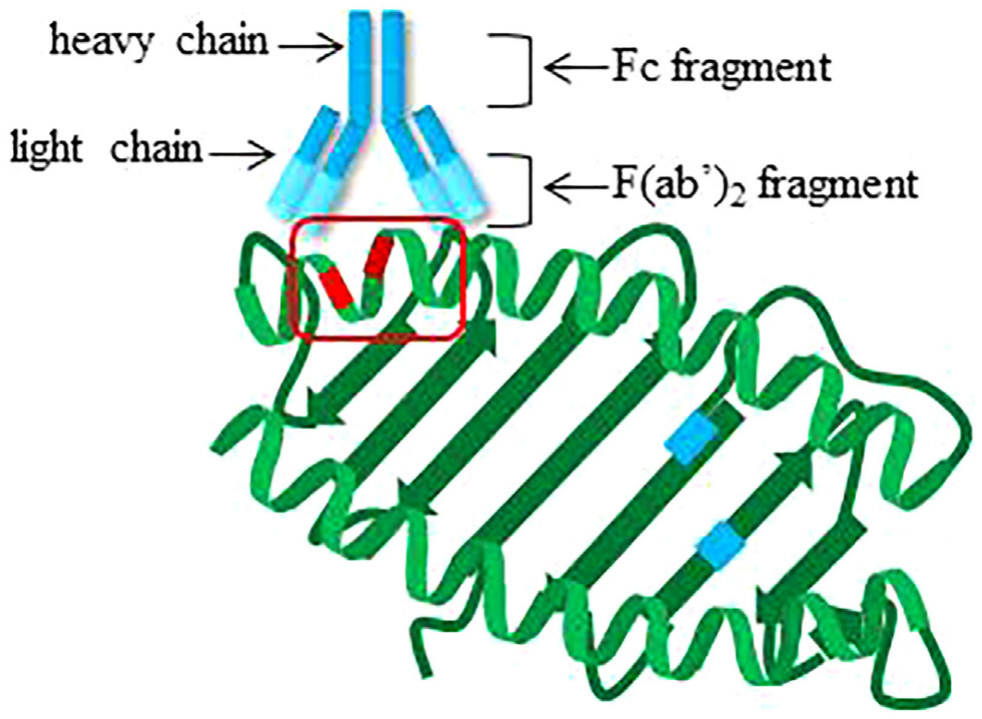
**Cartoon structure of antibody engaging epitope on HLA class I**. Theoretical locations of polymorphisms impacting peptide binding are indicated in blue on the beta sheet of HLA class I. Locations of polymorphic amino acid residues available for recognition by antibodies on the alpha helices are highlighted in red. *Red box*, epitope.

Alloantibodies can be generated against any of the polymorphic loci, i.e., HLA-A, -B, -Cw, DRB1, DRB3 (DR52), DRB4 (DR53), DRB5 (DR51), DQB1, DQA1, DPB1, and DPA1. Antibodies recognize three-dimensional arrangements of amino acids on antigens, called epitopes. Fifteen to 25 amino acid residues form epitopes that are not necessarily adjacent in linear sequence, but are generally within 4 Å (44) (Figure 3). Many of the amino acid polymorphisms within HLA molecules lie within and around the peptide-binding groove at exposed residues on the alpha helices of the α1 and α2 chains of HLA class I, and on the α1 and β1 chains of HLA class II, enabling presentation of diverse peptides. The host–pathogen arms race is believed to have driven this polymorphism to prevent pathogen immune escape and protect populations from epidemics (45). Interestingly, antibody reactivity may also be influenced by the bound peptide (46), which can alter the overall three-dimensional conformation of HLA.

### Immunogenicity of HLA

Alloantibodies recognize three-dimensional amino acid epitopes on non-self HLA molecules. Because of the evolution of the HLA system, these epitopes can be shared by many different antigens, leading to broad antibody sensitization after exposure to a limited repertoire of non-self HLA. Antibodies are also sensitive enough to recognize single amino acid differences, resulting in intra-allele antibody production (47). Furthermore, molecular differences between HLA antigens can affect expression levels at the surface of the cell. Below, we describe in greater detail the mechanisms governing these various aspects of alloantibody recognition of epitopes.

#### Cross-Reactive Antibody Groups

The diversity of HLA has been driven by several genetic processes during positive selection. One major mechanism is gene conversion via homologous recombination. Gene conversion results in large segments of genetic material being shared between alleles, giving rise to multiple proteins with the same or similar amino acid epitopes that can be recognized by the alloantibody response (48). This epitope sharing also results in cross-reactive antibody groups (CREGs), and indeed phylogenetic grouping of HLA based on nucleotide sequences generally mirrors serological cross-reactivity (49). Broad sensitization against many HLA antigens can, thus, occur even when the immune system is only exposed to a single non-self HLA antigen. For example, exposure to HLA-A11 may result in the generation of an antibody that is specific to an epitope carried by multiple HLA antigens belonging to the A1 CREG including HLA-A1, A3, A23, A24, A36, and A80 as well as A11 (50, 51). In another example demonstrating inter-locus reactivity, sensitization to HLA-Cw can lead to antibody production to antigens of HLA-B (52) as HLA-B and HLA-Cw are more closely related to one another than to HLA-A. Similarly, DP antigens share epitopes with DR (53).

An extreme example of broad sensitization is in response to the mutually exclusive public epitopes Bw4 and Bw6, which are present on many different HLA-B (as well as some HLA-A, for Bw4) antigens. An individual exposed to a Bw6 positive antigen, such as B7, may produce antibodies against the Bw6 epitope that react with more than 20 different HLA-B antigens, carried by more than 50% of the population. These determinants, as well as C1 and C2 determinants on HLA-Cw molecules, are critical for NK cell receptor (KIR) binding, and so have likely been conserved through co-evolution of HLA and KIR receptors to prevent loss of self recognition (54).

In seminal work, Parham and McLean (55) described serological reactivity in relationship to known amino acid sequence data, first raising the idea of molecular matching. Differences in epitopes or “structural compatibility” between self and potential donor HLA antigens, also known as “eplets,” could portend the likelihood of an antibody response. HLA typing for solid organ transplantation is generally reported at the serologic (two digit) level. However, epitope matching is best accomplished with higher resolution HLA typing such that amino acid sequences that may be different within serologically equivalent groups are defined. Several groups have advocated for the use of structural epitope or eplet matching strategies in organ allocation, over serologic level matching (56, 57). For example, Wiebe et al. reported a lower incidence of *de novo* DSA production in patients who were HLA class II epitope matched (58), and immunogenicity of HLA-DP (59) also appears to be strongly based on epitope recognition.

#### Allele-Specific Antibodies

Antibodies can be produced against epitopes within antigens that differ from self by as little as one amino acid. Therefore, in addition to antibodies against serologic level HLA molecules, individuals can produce antibodies to other alleles of “self” antigens, if amino acid sequences in key positions are sufficiently disparate. For example, a patient who displays HLA-DQ6 at the serologic level may also be defined through higher resolution typing methods as DQB1*06:01 at the allele level. The patient may become sensitized to other alleles of DQ6 and display allele-specific antibodies to alleles, such as DQB1*06:04, that are distinct from self (60).

#### Epitopes Formed by Specific DQA1/DQB1 Pairings

It is also possible for individuals to make antibodies against an epitope that is formed by the pairing of specific DQα1 and DQβ1 chains (61). The majority of HLA-DQ reactive antibodies recognize the DQβ chain, while a minority (<20%) bind DQα chain or a combination epitope formed by specific DQα/β pairings (61). Importantly, such antibodies do not produce positive crossmatches against donors who carry only one of the DQα or DQβ alleles in a different pairing (62), emphasizing the specificity of such antibodies.

#### Molecular Contributions to Immunogenicity

Differences in antigen availability may necessarily influence immunogenicity. Cell surface expression levels are known to vary among different loci, and even different alleles, of HLA. Certainly, expression of HLA-Cw (63, 64) and HLA-DP (65) is less abundant than proteins of other loci on endothelial cells that make up the vascular walls of the transplanted organ. In addition, HLA-A was found to be more highly expressed than HLA-B in HEK293T cells as it is hypothesized to form a more stable interaction with β2m throughout the terminal region of the alpha 2 domain and the entire alpha 3 domain (66) of the molecules.

Furthermore, Ramsuran et al. recently reported wide variation in mRNA levels between different antigens of HLA-A; for example, individuals homozygous for HLA-A24 had higher expression of HLA-A than those homozygous for HLA-A3, which was attributed to polymorphic CpG sites and increased DNA methylation in the lower expressing alleles (67). Finally, lower expression of HLA-Cw may be the result of reduced affinity for β2 microglobulin, resulting in less stable protein at the cell surface (68, 69), increased degradation of mRNA (63), or differential regulation by miRNA (70). Accordingly, sensitization to HLA-Cw is reportedly less frequent compared with other HLA class I molecules (71).

## Sensitizing Events Leading to HLA Immunization: Routes and Rates of HLA Sensitization

Antibody responses to allogeneic HLA molecules can occur after any exposure to non-self tissues, such as transfusion, pregnancy, or transplantation. However, the durability and nature of the sensitization may vary depending on the alloimmunizing event.

### Transfusion

Interestingly, the incidence of alloimmunization in the general population with a history of prior transfusion is less than 2% (72, 73), while in comparison Hyun et al. (74) reported that one-third of transplant candidates with a history of transfusion were sensitized. The discrepancy indicates that transplant patients may have a more robust response to sensitization via transfusion, or may have more transfusions compared with non-transplant candidates.

Transfusion alone is considered poorly immunogenic. Sensitization to HLA antigens via transfusion requires very large blood volumes or multiple events to induce persistent HLA allosensitization in otherwise non-sensitized individuals (34). Paradoxically, a protective “transfusion effect” was reported in the early transplantation literature (75, 76), initially suggesting that donor-specific transfusion is immunomodulatory and improved graft outcomes. Animal models have suggested that graft passenger leukocytes are important in this process, thus, providing tolerance prior to transplant (77). However, transplant recipients sensitized by third party transfusion have poorer 1-year survival compared with non-sensitized recipients (78). A modern meta-analysis of that era concluded that higher rates of HLA sensitization are found in patients with a history of transfusion compared with those without, and that there is a neutral to negative effect on allograft outcome after sensitization by transfusion (79).

### Pregnancy

Both full-term pregnancy and spontaneous miscarriage induce alloantibodies (80). Anti-paternal alloantibodies appear around or after the 28th week of gestation during pregnancy (80). Sensitized women have higher rates of parity (pregnancy) compared with non-sensitized patients (81). One-third to half of women develop HLA immunization after delivery during their first pregnancy (73, 74, 82), and immunization frequency increases with parity (82). Antibodies to HLA class I were slightly more frequent than those to class II, although both were produced.

Female patients receiving kidney allografts from their male partners or their offspring experienced higher rejection rates (83), pointing to increased immunological risk in women upon re-exposure to paternal antigens on the allograft. Generally, antibodies induced by pregnancy declined in the circulation over time. Even so, post-transplant antibody increases occurred in the pregnancy cohort even decades after the last pregnancy (84).

### Transplantation

Transplantation itself is a significant alloimmunizing event (81), and previously non-donor sensitized solid organ transplant recipients develop *de novo* donor-specific HLA antibodies at a rate of about 8–10% in the first year for liver and renal transplants (85, 86), and 15–25% of renal and cardiac transplant by 10 years post-transplant (85, 87, 88). Removal (transplantectomy) of failed renal-allografts appears to stimulate a large increase in circulating DSA (89), whether from increased immune activation in response to surgical trauma, removal of the antigen “sink” provided by the allograft and/or immunosuppression, is unclear.

When evaluating a patient for re-transplantation, it is important to consider the presence of donor-specific alloantibodies that were formed via sensitization to the first allograft in relationship to the donor antigens carried by the second potential donor – the so-called “repeat mismatches.” Repeat mismatched donor HLA antigens against which a recipient has preformed alloantibody, particularly to HLA-DR, were found to have a detrimental effect on renal-allograft survival (90–92). While Farney et al. did not uncover a deleterious effect on graft survival of retransplantation with donors who shared mismatches in the presence of alloantibodies with prior donors (93), a more recent study found that re-exposure to mismatched HLA class I antigens increased the risk of early graft loss in renal transplant recipients (94). Typically, repeat HLA mismatches in donors against which a recipient has made antibodies are avoided by transplant programs (95).

Allografts are also used for vascular reconstruction in many forms of congenital heart disease and have been demonstrated to cause persistent sensitization to HLA antigens (96). These findings have implications for those in whom heart transplant is considered late in the clinical course.

### Ventricular Assist Devices

Ventricular Assist Devices (VADs) are associated with increased production of HLA antibodies. The current paradigm is that the VAD provides a continual antigenic or inflammatory stimulus that promotes generation of *de novo* HLA antibodies when patients are exposed to blood and/or platelet transfusions or heightens existing HLA antibody levels. In a recent study, we observed that patients implanted with the older pulsatile VAD (BiVad) showed increased HLA sensitization vs. patients implanted with the Heart MateII Axial VAD suggesting the older, pulsatile devices had greater sensitization potential (97).

### Natural Antibodies

It has been suggested that HLA antibodies may be formed by means other than the typical routes of sensitization discussed above. Antibodies to HLA are found in non-transfused males at a rate of nearly 50% in one study (98) and often react with a restricted subset of HLA antigens that are uncommon in the general population. There is some evidence pointing to cross-reactivity of pathogens (especially viral) with HLA by T cells (99–102). The abundant viral-specific memory T cell repertoire may, therefore, contribute to alloantibody production. Viral cross-reactivity with HLA may also occur at the protein level. For example, antibodies to HIV-1 may recognize HLA (103), and immunization with the HepB vaccine caused HLA antibody positivity in approximately half of previously negative, healthy adults 1 month after vaccination (104).

It has been proposed that “natural antibodies” against the non-classical HLA-E molecule can cross-react with HLA class I molecules (105). Alternatively, it is possible that antibodies detected are false-positive reactions with denatured antigen, a known limitation of the single-antigen bead assay commonly used to identify HLA antibodies in the sera (106–108). Additional evidence shows these antibodies do not often react with native antigen on cells (108, 109), and the clinical significance and durability of such natural antibody responses remain unclear (110, 111).

### Non-HLA Antibodies

Non-HLA antibodies can be directed toward either alloantigens, such as the major histocompatibility complex class I chain-related gene A (MICA) or B (MICB), or tissue-specific autoantigens, such as vimentin, cardiac myosin (CM), collagen V (Col V), agrin and angiotensin II receptor type I (AT1R). Additional non-HLA targets recently identified by Jackson et al. include anti-endothelial cell targets, including endoglin, EGF-like repeats, Fms-like tyrosine kinase-3 ligand, and ICAM-4. The principle antigenic targets of non-HLA antibodies are expressed on cells of the allograft, including endothelium and epithelium. Therefore, donor cells are in direct contact with the recipients circulating peripheral blood lymphocytes, and have been shown to be the major immunological targets for the pathogenesis of allograft rejection. Prevalence of anti-endothelial cell antibodies (AECA) among renal recipients was nearly one quarter in pre-transplant sera (112). AECAs correlated with post-transplant HLA DSA and AMR. Sun et al. observed that anti-endothelial cell antibodies were found in patients pre-transplant, but that they did not correlate with outcome or rejection; by contrast, *de novo* development of AECAs was significantly associated with early and severe acute rejection, but not C4d (113). AECA were implicated as the cause of acute antibody-mediated rejection (AMR) in 30% of heart transplant recipients without DSA to HLA (114).

## Mechanisms of Graft Damage by HLA Antibodies

High-titered pre-transplant DSA directed against HLA class I antigens can cause catastrophic hyperacute rejection and immediate graft loss (115), whereas high titer class II DSA mediate graft rejection 2–4 days after transplant, upon re-expression of HLA class II antigens on the endothelium of the allograft (116–118). By contrast, pre-transplant DSA of low titer are often associated with development of acute AMR during the first 3 months after transplantation and/or lower long-term graft survival (119). If left untreated, patients with AMR are at risk of graft loss and/or markedly shortened overall graft survival time. Patients producing *de novo* anti-HLA antibodies against their donor following transplantation are also at increased risk of graft failure unless their response can be controlled or abrogated (120).

There are three major effector functions carried out by antibodies that can impact the graft. First, bivalent IgG can dimerize or crosslink its target upon binding. Collective studies indicate that IgG binding to HLA agonistically crosslinks HLA molecules and triggers downstream activation of the target cells. Second, antibodies can activate the classical complement cascade through binding to the Fc fragment to trigger production of potent anaphylatoxins, chemoattractants, opsonins, and cell-damaging factors. Thirdly, HLA IgG bound to target cells can engage Fc receptors on myeloid and lymphoid cells, to employ a host of Fc receptor-mediated effector functions, including antibody-dependent cell cytotoxicity, antibody-dependent phagocytosis, and augment recruitment. These effector functions work in concert, and there is substantial interplay between them, as we will discuss below.

### HLA Antibody-Induced Signaling in Graft Vascular Cells

Antibodies are capable of agonistically crosslinking their protein targets at the cell surface [recently reviewed in Ref. (121)]. In vascular cells, crosslinking of HLA induces intracellular signaling cascades that lead to functional changes, such as increased cell migration, cytoskeletal rearrangement, growth and proliferation, endothelial activation and exocytosis, and increased recruitment of leukocytes. These functional changes parallel the histological findings in clinical AMR, including microvascular inflammation, endothelial dysfunction, expansion of the neointima, and infiltration of mononuclear cells (Table 2).

**Table 2:** **Function of HLA antibodies leading to histological manifestations of AMR**.

Histological manifestation	Antibody function
C4d deposition	Activation of complement
Endothelial cell swelling	HLA crosslinking leading to cytoskeletal changes
Mononuclear cell infiltration	HLA crosslinking increases P-selectin and chemokines, monocyte, and neutrophil adherence
	Antibody Fc regions interact with FcγRs
Neointimal thickening	HLA crosslinking increases endothelial and smooth muscle cell proliferation and migration

HLA class I and II do not have intrinsic kinase activity and, therefore, partner with other proteins to transduce intracellular signals. Ligation of HLA class I with antibodies increases its association with integrin β4 which, in turn, activates intracellular signaling cascades (122). Integrin β4 is an important cell adhesion protein regulating cell adhesion, proliferation, ­migration, and survival. Blockade of integrin β4 impairs HLA antibody-stimulated signal transduction. Protein(s) that partner with HLA class II to transduce signaling are not yet reported. However, ligation of either HLA class I or class II with antibodies activates mammalian target of rapamycin (mTOR) signaling through the SRC/FAK–PI3K–AKT pathway and increases Akt-dependent cell survival signaling, through upregulation of Bcl-2 and HO-1 (123–125). Furthermore, activation of key signaling proteins in endothelium, including S6K, S6RP, and ERK, demonstrated in human cardiac allografts with AMR and in murine models of MHC antibody-mediated injury (126–128) suggests pro-survival signals that may increase endothelial persistence, stress fiber formation (129, 130), and resistance to complement-induced cell death (125), contributing to neointimal formation during chronic rejection. Additional work by Galvani et al. points to a direct effect of MHC antibodies on smooth muscle cells. Crosslinking of HLA I with antibodies provokes mitogenic signaling through matrix metalloproteinases *in vitro*, and contributes to neointimal thickening of human arterial grafts *in vivo* in murine recipients (131, 132). An additional feature of alloantibody crosslinking of HLA is increased intracellular calcium levels, leading to exocytosis of endothelial Weibel–Palade body vesicles and increased cell surface P-selectin (133–135). P-selectin captures neutrophils and monocytes (133, 134), facilitating recruitment of immune cells into the allograft.

### Complement

The complement system is an ancient form of innate immunity that relies on proteolytic cleavage of active components. Complement proteins are always present in the circulation, but become rapidly activated upon exposure to target molecules. There are three main pathways of complement, which differ by the activating stimulus. The lectin pathway becomes activated upon recognition by mannose binding lectin (MBL) of pathogen-specific glycan residues on the surface of bacteria, fungi, and viruses. The alternative pathway of complement is initiated at the surface of non-host cells due to the presence of such factors as lipopolysaccharides on Gram-negative bacteria, zymosans on fungi and yeast, and other pathogen-associated molecules. Complexed human immunoglobulin has also been shown to activate the alternative pathway. The classical complement pathway is initiated exclusively by antigen-bound antibody through binding of the Fc portion of certain isotypes and subclasses to C1q. All of these pathways rely on sequential enzymatic reactions that produce active split products involved in inflammation, and all of these pathways converge on the terminal component C5.

Activation of complement by antibodies was one of the earliest methods used to detect donor-specific HLA antibodies, and positive cytotoxic crossmatch is still often considered to be a contraindication to transplant, as antibodies detected by this method can mediate hyperacute rejection of solid organ transplants (115). Although the end result of complement activation, namely deposition of MAC and cell cytotoxicity, has been a focus, it is now thought to be a rare event (136). Endothelial cells express complement regulatory proteins (CD55/DAF, CD59, Crry) that antagonize complement activation by inactivating split products. C3d and C4d are generated by such inhibitory receptors and mark early complement activation. Attention has turned to the activity and predictive value of other complement proteins. Products of complement activation, in particular C4d, have proven histological utility in detecting donor-specific antibody bound to the graft (137, 138). Other split products, including C4a, C3a, and C3b, are potent inflammatory signals that promote immune cell recruitment and opsonization.

### FcγR-Bearing Immune Cells

Many cells express surface receptors that can interact with the constant region heavy chain (Fc) of antibodies. The human Fc receptor system consists of several classes that can bind to IgG (FcγR, CD64, CD32, CD16), IgA (FcαR, CD89), and IgE (FcϵR, CD23). The human receptor for IgM (FcμR) had been elusive until relatively recently (139). Fc receptors serve to bridge the humoral and cellular arms of the immune system, and provide innate immune cells with a target, and are critical for a variety of functions, including antibody-dependent cell-mediated phagocytosis (ADCP), antibody-dependent cell-mediated cytotoxicity (ADCC), cell–cell tethering and degranulation.

Given that IgG is thought to be the most clinically relevant isotype of HLA antibodies, we will focus on Fc-gamma receptors (FcγR) that bind to this isotype of immunoglobulins. FcγRs are expressed broadly in both the myeloid and lymphoid compartments. There are three major classes of FcγRs, FcγRI (CD64), FcγRII (CD32), and FcγRIII (CD16). FcγRII and FcγRIII are further composed of several functionally disparate isoforms, most of which are dimorphic in the human population (140, 141). Polymorphisms in human FcγRs influence susceptibility to autoimmune disease and response to anti-tumor therapeutics (142–146), and may also influence susceptibility of transplant recipients to rejection (147, 148), although a thorough evaluation of the role of different FcγR alleles in antibody-mediated transplant rejection has not been reported.

Due to their lower affinity, the majority of FcγRs do not bind monomeric IgG very efficiently. Only the high-affinity FcγRI (CD64) is the exception, and cells with this receptor have been shown to carry monomeric IgG in circulation. FcγRs do bind to antigen-associated IgG, however, such as in immune complexes or immobilized on a (cell) surface. Once bound, FcγRs become crosslinked as they physically colocalize at high antibody-antigen density. This promotes intracellular signaling in the FcγR-bearing cell leading to activation and maturation, and mediates effector functions such as phagocytosis or cytotoxicity.

The relevance of FcγR-bearing innate immune cells to antibody-mediated graft injury is reflected in the diagnostic criteria and histological manifestations of AMR. For example, infiltration of CD68+ macrophages is included in the AMR diagnostic criteria in cardiac transplantation (149), where macrophage staining is found intravascularly (150, 151). Indeed, increased macrophage burden is correlative with worse prognosis (152). Although not currently included in the AMR diagnostic criteria for renal transplantation, macrophage infiltration during rejection is also predictive of worse outcome in kidney allografts (153, 154). Our recent studies are consistent with these clinical findings and show that monocyte recruitment to HLA-Ab-activated endothelium is mediated by HLA-induced Weibel–Palade exocytosis and P-selectin expression (134). Blockade of P-selectin potently inhibited leukocyte recruitment to the allograft during AMR underscoring its therapeutic potential (134). Furthermore, HLA-Ab augmented monocyte recruitment by the interaction of monocyte FcγRs with the Fc portion of the HLA-Abs (135). This interaction was IgG subclass dependent and influenced by monocyte FcγRIIa allelic variants. Monocytes from donors carrying the high-affinity FcγRIIa-H131 allele had greater FcγR-dependent adhesion to ECs activated with HLA-Abs of both IgG1 and IgG2 subclasses compared with monocytes expressing only FcγRIIa-R131. These results are clinically relevant and suggest that recipients producing DSA and carrying high-affinity FcγR alleles may be pre-disposed to acute AMR accompanied by increased monocyte infiltration.

### Summary

Taken together, antibodies to donor proteins, including HLA, can cause graft damage through three major mechanisms, including direct activation of endothelial, smooth muscle, and epithelial cells to promote proliferation and inflammation; activation of the complement system to generate inflammatory split products; and engagement of FcγRs on NK cells, monocytes, and neutrophils.

## Conclusion

Allorecognition by the humoral immune system results in formation of antibodies to HLA and a variety of non-HLA proteins, and occurs after exposure to non-self tissues through pregnancy, transfusion, or transplantation. Alloantibody formation is dependent upon T cell interactions and is primarily driven by indirect allorecognition by T cells. In addition, “natural” antibodies or anti-viral antibodies may cross-react with HLA, although the clinical significance of such antibodies is not clear. Antibodies to donor HLA mediate allograft injury through Fc-dependent as well as Fc-independent mechanisms, which closely reflect the diagnostic criteria for AMR. Non-HLA antibodies can be against polymorphic proteins, such as MICA, or against autoantibodies, and also associate with worse graft outcome, although their etiology is less clear than for HLA DSA.

## Author Contributions

MH was responsible for outlining, research, and writing of the manuscript. NV was responsible for outlining, research, and writing of the manuscript. ER reviewed and critically examined the manuscript and shaped its final version.

## Conflict of Interest Statement

The authors declare that the research was conducted in the absence of any commercial or financial relationships that could be construed as a potential conflict of interest.

## References

[1] Steele DJ Laufer TM Smiley ST Ando Y Grusby MJ Glimcher LH Two levels of help for B cell alloantibody production. J Exp Med (1996) 183(2):699–703. 10.1084/jem.183.2.699 8627185 PMC2192460

[2] Afzali B Lombardi G Lechler RI. Pathways of major histocompatibility complex allorecognition. Curr Opin Organ Transplant (2008) 13(4):438–44. 10.1097/MOT.0b013e328309ee31 18685342 PMC3815495

[3] Zhang JQ HM Valenzuela NM Zhang X Lan JH Cecka M Reed EF Histocompatibility and Immunogenetics for Solid Organ Transplantation. London: Springer Science+Business Media (2015).

[4] IMGT/HLA. Available from: http://www.ebi.ac.uk/ipd/imgt/hla/stats.html.

[5] Taylor AL Negus SL Negus M Bolton EM Bradley JA Pettigrew GJ. Pathways of helper CD4 T cell allorecognition in generating alloantibody and CD8 T cell alloimmunity. Transplantation (2007) 83(7):931–7. 10.1097/01.tp.0000257960.07783.e3 17460565

[6] Conlon TM Saeb-Parsy K Cole JL Motallebzadeh R Qureshi MS Rehakova S Germinal center alloantibody responses are mediated exclusively by indirect-pathway CD4 T follicular helper cells. J Immunol (2012) 188(6):2643–52. 10.4049/jimmunol.1102830 22323543 PMC3378630

[7] Hornick PI Mason PD Yacoub MH Rose ML Batchelor R Lechler RI. Assessment of the contribution that direct allorecognition makes to the progression of chronic cardiac transplant rejection in humans. Circulation (1998) 97(13):1257–63. 10.1161/01.CIR.97.13.1257 9570195

[8] Benichou G Valujskikh A Heeger PS. Contributions of direct and indirect T cell alloreactivity during allograft rejection in mice. J Immunol (1999) 162(1):352–8. 9886406

[9] Hornick PI Mason PD Baker RJ Hernandez-Fuentes M Frasca L Lombardi G Significant frequencies of T cells with indirect anti-donor specificity in heart graft recipients with chronic rejection. Circulation (2000) 101(20):2405–10. 10.1161/01.CIR.101.20.2405 10821818

[10] Baker RJ Hernandez-Fuentes MP Brookes PA Chaudhry AN Cook HT Lechler RI. Loss of direct and maintenance of indirect alloresponses in renal allograft recipients: implications for the pathogenesis of chronic allograft nephropathy. J Immunol (2001) 167(12):7199–206. 10.4049/jimmunol.167.12.7199 11739543

[11] Richters CD van Gelderop E du Pont JS Hoekstra MJ Kreis RW Kamperdijk EW. Migration of dendritic cells to the draining lymph node after allogeneic or congeneic rat skin transplantation. Transplantation (1999) 67(6):828–32. 10.1097/00007890-199903270-00008 10199730

[12] Sandner SE Salama AD Houser SL Palmer E Turka LA Sayegh MH. New TCR transgenic model for tracking allospecific CD4 T-cell activation and tolerance in vivo. Am J Transplant (2003) 3(10):1242–50. 10.1046/j.1600-6143.2003.00220.X 14510697

[13] Kaminski E Hows J Man S Brookes P Mackinnon S Hughes T Prediction of graft versus host disease by frequency analysis of cytotoxic T cells after unrelated donor bone marrow transplantation. Transplantation (1989) 48(4):608–13. 2508280

[14] Schwarer AP Jiang YZ Deacock S Brookes PA Barrett AJ Goldman JM Comparison of helper and cytotoxic antirecipient T cell frequencies in unrelated bone marrow transplantation. Transplantation (1994) 58(11):1198–203. 10.1097/00007890-199412270-00011 7992363

[15] Lechler RI Batchelor JR. Immunogenicity of retransplanted rat kidney allografts. Effect of inducing chimerism in the first recipient and quantitative studies on immunosuppression of the second recipient. J Exp Med (1982) 156(6):1835–41. 10.1084/jem.156.6.1835 6757374 PMC2186859

[16] Braun MY McCormack A Webb G Batchelor JR Mediation of acute but not chronic rejection of MHC-incompatible rat kidney grafts by alloreactive CD4 T cells activated by the direct pathway of sensitization. Transplantation (1993) 55(1):177–82. 10.1097/00007890-199301000-00033 8093565

[17] Lee RS Grusby MJ Glimcher LH Winn HJ Auchincloss H Jr. Indirect recognition by helper cells can induce donor-specific cytotoxic T lymphocytes in vivo. J Exp Med (1994) 179(3):865–72. 10.1084/jem.179.3.865 8113680 PMC2191395

[18] Wise MP Bemelman F Cobbold SP Waldmann H. Linked suppression of skin graft rejection can operate through indirect recognition. J Immunol (1998) 161(11):5813–6. 9834057

[19] Bennett SR Carbone FR Karamalis F Miller JF Heath WR. Induction of a CD8+ cytotoxic T lymphocyte response by cross-priming requires cognate CD4+ T cell help. J Exp Med (1997) 186(1):65–70. 10.1084/jem.186.1.65 9206998 PMC2198964

[20] Ridge JP Di Rosa F Matzinger P. A conditioned dendritic cell can be a temporal bridge between a CD4+ T-helper and a T-killer cell. Nature (1998) 393(6684):474–8. 10.1038/30989 9624003

[21] Schoenberger SP Toes RE van der Voort EI Offringa R Melief CJ. T-cell help for cytotoxic T lymphocytes is mediated by CD40-CD40L interactions. Nature (1998) 393(6684):480–3. 10.1038/31002 9624005

[22] Smyth LA Afzali B Tsang J Lombardi G Lechler RI Intercellular transfer of MHC and immunological molecules: molecular mechanisms and biological significance. Am J Transplant (2007) 7(6):1442–9. 10.1111/j.1600-6143.2007.01816.x 17511673 PMC3815510

[23] Herrera OB Golshayan D Tibbott R Salcido Ochoa F James MJ Marelli-Berg FM A novel pathway of alloantigen presentation by dendritic cells. J Immunol (2004) 173(8):4828–37. 10.4049/jimmunol.173.8.4828 15470023

[24] Smyth LA Herrera OB Golshayan D Lombardi G Lechler RI. A novel pathway of antigen presentation by dendritic and endothelial cells: implications for allorecognition and infectious diseases. Transplantation (2006) 82(1 Suppl):S15–8. 10.1097/01.tp.0000231347.06149.ca 16829787

[25] Curry AJ Pettigrew GJ Negus MC Easterfield AJ Young JL Bolton EM Dendritic cells internalise and re-present conformationally intact soluble MHC class I alloantigen for generation of alloantibody. Eur J Immunol (2007) 37(3):696–705. 10.1002/eji.200636543 17266175

[26] McHeyzer-Williams LJ Pelletier N Mark L Fazilleau N McHeyzer-Williams MG. Follicular helper T cells as cognate regulators of B cell immunity. Curr Opin Immunol (2009) 21(3):266–73. 10.1016/j.coi.2009.05.010 19502021 PMC2731669

[27] Qi H Cannons JL Klauschen F Schwartzberg PL Germain RN. SAP-controlled T-B cell interactions underlie germinal centre formation. Nature (2008) 455(7214):764–9. 10.1038/nature07186 18843362 PMC2652134

[28] Cannons JL Qi H Lu KT Dutta M Gomez-Rodriguez J Cheng J Optimal germinal center responses require a multistage T cell: B cell adhesion process involving integrins, SLAM-associated protein, and CD84. Immunity (2010) 32(2):253–65. 10.1016/j.immuni.2010.01.010 20153220 PMC2830297

[29] Allen CD Okada T Cyster JG. Germinal-center organization and cellular dynamics. Immunity (2007) 27(2):190–202. 10.1016/j.immuni.2007.07.009 17723214 PMC2242846

[30] Allen CD Okada T Tang HL Cyster JG. Imaging of germinal center selection events during affinity maturation. Science (2007) 315(5811):528–31. 10.1126/science.1136736 17185562

[31] Phan TG Paus D Chan TD Turner ML Nutt SL Basten A High affinity germinal center B cells are actively selected into the plasma cell compartment. J Exp Med (2006) 203(11):2419–24. 10.1084/jem.20061254 17030950 PMC2118125

[32] Maruyama M Lam KP Rajewsky K. Memory B-cell persistence is independent of persisting immunizing antigen. Nature (2000) 407(6804):636–42. 10.1038/35036600 11034213

[33] Snanoudj R Claas FH Heidt S Legendre C Chatenoud L Candon S. Restricted specificity of peripheral alloreactive memory B cells in HLA-sensitized patients awaiting a kidney transplant. Kidney Int (2015) 87(6):1230–40. 10.1038/ki.2014.390 25565312

[34] Scornik JC Meier-Kriesche HU. Blood transfusions in organ transplant patients: mechanisms of sensitization and implications for prevention. Am J Transplant (2011) 11(9):1785–91. 10.1111/j.1600-6143.2011.03705.x 21883910

[35] Bestard O Crespo E Stein M Lucia M Roelen DL de Vaal YJ Cross-validation of IFN-gamma Elispot assay for measuring alloreactive memory/effector T cell responses in renal transplant recipients. Am J Transplant (2013) 13(7):1880–90. 10.1111/ajt.12285 23763435

[36] Hricik DE Rodriguez V Riley J Bryan K Tary-Lehmann M Greenspan N Enzyme linked immunosorbent spot (ELISPOT) assay for interferon-gamma independently predicts renal function in kidney transplant recipients. Am J Transplant (2003) 3(7):878–84. 10.1034/j.1600-6143.2003.00132.x 12814480

[37] de Haan A van den Berg AP Hepkema BG van Dijk E Haagsma EB The TH Donor-specific hyporeactivity after liver transplantation: prominent decreases in donor-specific cytotoxic T lymphocyte precursor frequencies independent of changes in helper T lymphocyte precursor frequencies or suppressor cell activity. Transplantation (1998) 66(4):516–22. 10.1097/00007890-199808270-00017 9734497

[38] Vella JP Spadafora-Ferreira M Murphy B Alexander SI Harmon W Carpenter CB Indirect allorecognition of major histocompatibility complex allopeptides in human renal transplant recipients with chronic graft dysfunction. Transplantation (1997) 64(6):795–800. 10.1097/00007890-199712270-00033 9326400

[39] Liu Z Colovai AI Tugulea S Reed EF Fisher PE Mancini D Indirect recognition of donor HLA-DR peptides in organ allograft rejection. J Clin Invest (1996) 98(5):1150–7. 10.1172/JCI118898 8787678 PMC507537

[40] Jackson KJ Wang Y Collins AM. Human immunoglobulin classes and subclasses show variability in VDJ gene mutation levels. Immunol Cell Biol (2014) 92(8):729–33. 10.1038/icb.2014.44 24913324

[41] van Zelm MC B cells take their time: sequential IgG class switching over the course of an immune response? Immunol Cell Biol (2014) 92(8):645–6. 10.1038/icb.2014.48 24935459

[42] Vidarsson G Dekkers G Rispens T. IgG subclasses and allotypes: from structure to effector functions. Front Immunol (2014) 5:520. 10.3389/fimmu.2014.00520 25368619 PMC4202688

[43] Devey ME Bleasdale-Barr KM Bird P Amlot PL. Antibodies of different human IgG subclasses show distinct patterns of affinity maturation after immunization with keyhole limpet haemocyanin. Immunology (1990) 70(2):168–74. 2373517 PMC1384188

[44] Duquesnoy RJ. A structurally based approach to determine HLA compatibility at the humoral immune level. Hum Immunol (2006) 67(11):847–62. 10.1016/j.humimm.2006.08.073 17145365 PMC2169290

[45] Hertz T Nolan D James I John M Gaudieri S Phillips E Mapping the landscape of host-pathogen coevolution: HLA class I binding and its relationship with evolutionary conservation in human and viral proteins. J Virol (2011) 85(3):1310–21. 10.1128/JVI.01966-10 21084470 PMC3020499

[46] Mulder A Eijsink C Kester MG Franke ME Kardol MJ Heemskerk MH Impact of peptides on the recognition of HLA class I molecules by human HLA antibodies. J Immunol (2005) 175(9):5950–7. 10.4049/jimmunol.175.9.5950 16237088

[47] Nakayama S Kawaguchi G Karaki S Nagao T Uchida H Kashiwase K Effect of single amino acid substitution at residue 167 of HLA-B51 on binding of antibodies and recognition of T cells. Hum Immunol (1994) 39(3):211–9. 10.1016/0198-8859(94)90262-3 8026989

[48] Rodey GE Neylan JF Whelchel JD Revels KW Bray RA. Epitope specificity of HLA class I alloantibodies. I. Frequency analysis of antibodies to private versus public specificities in potential transplant recipients. Hum Immunol (1994) 39(4):272–80. 10.1016/0198-8859(94)90270-4 7520897

[49] McKenzie LM Pecon-Slattery J Carrington M O’Brien SJ. Taxonomic hierarchy of HLA class I allele sequences. Genes Immun (1999) 1(2):120–9. 10.1038/sj.gene.6363648 11196658

[50] Wade JA Hurley CK Takemoto SK Thompson J Davies SM Fuller TC HLA mismatching within or outside of cross-reactive groups (CREGs) is associated with similar outcomes after unrelated hematopoietic stem cell transplantation. Blood (2007) 109(9):4064–70. 10.1182/blood-2006-06-032193 17202313 PMC1874562

[51] Marrari M Mostecki J Mulder A Claas F Balazs I Duquesnoy RJ. Human monoclonal antibody reactivity with human leukocyte antigen class I epitopes defined by pairs of mismatched eplets and self-eplets. Transplantation (2010) 90(12):1468–72. 10.1097/TP.0b013e3182007b74 21063243

[52] Lomago J Jelenik L Zern D Howe J Martell J Zeevi A How did a patient who types for HLA-B*4403 develop antibodies that react with HLA-B*4402? Hum Immunol (2010) 71(2):176–8. 10.1016/j.humimm.2009.11.013 19963027

[53] Billen EV Christiaans MH Doxiadis II Voorter CE van den Berg-Loonen EM. HLA-DP antibodies before and after renal transplantation. Tissue Antigens (2010) 75(3):278–85. 10.1111/j.1399-0039.2009.01428.x 20070601

[54] Norman PJ Hollenbach JA Nemat-Gorgani N Guethlein LA Hilton HG Pando MJ Co-evolution of human leukocyte antigen (HLA) class I ligands with killer-cell immunoglobulin-like receptors (KIR) in a genetically diverse population of sub-Saharan Africans. PLoS Genet (2013) 9(10):e1003938. 10.1371/journal.pgen.1003938 24204327 PMC3814319

[55] Parham P McLean J. Characterization, evolution, and molecular basis of a polymorphic antigenic determinant shared by HLA-A and B products. Hum Immunol (1980) 1(2):131–9. 10.1016/0198-8859(80)90100-7 6167545

[56] Tambur AR Buckingham M McDonald L Luo X. Development of ­donor-specific and non-donor-specific HLA-DP antibodies post-transplant: the role of epitope sharing and epitope matching. Clin Transpl (2006):399–404. 18365396

[57] Claas FH Rahmel A Doxiadis II Enhanced kidney allocation to highly sensitized patients by the acceptable mismatch program. Transplantation (2009) 88(4):447–52. 10.1097/TP.0b013e3181b04a5f 19696624

[58] Wiebe C Pochinco D Blydt-Hansen TD Ho J Birk PE Karpinski M Class II HLA epitope matching-A strategy to minimize de novo ­donor-specific antibody development and improve outcomes. Am J Transplant (2013) 13(12):3114–22. 10.1111/ajt.12478 24164958

[59] Laux G Mansmann U Deufel A Opelz G Mytilineos J. A new epitope-based HLA-DPB matching approach for cadaver kidney retransplants. Transplantation (2003) 75(9):1527–32. 10.1097/01.TP.0000061759.57702.8A 12792509

[60] Muro M Gonzalez-Soriano MJ Salgado G Lopez R Boix F Lopez M Specific “intra-allele” and “intra-broad antigen” human leukocyte antigen alloantibodies in kidney graft transplantation. Hum Immunol (2010) 71(9):857–60. 10.1016/j.humimm.2010.05.018 20510320

[61] Tambur AR Leventhal JR Friedewald JJ Ramon DS. The complexity of human leukocyte antigen (HLA)-DQ antibodies and its effect on virtual crossmatching. Transplantation (2010) 90(10):1117–24. 10.1097/00007890-201007272-00536 20847715

[62] Tambur AR Leventhal JR Zitzner JR Walsh RC Friedewald JJ. The DQ barrier: improving organ allocation equity using HLA-DQ information. Transplantation (2013) 95(4):635–40. 10.1097/TP.0b013e318277b30b 23288109

[63] McCutcheon JA Gumperz J Smith KD Lutz CT Parham P. Low HLA-C expression at cell surfaces correlates with increased turnover of heavy chain mRNA. J Exp Med (1995) 181(6):2085–95. 10.1084/jem.181.6.2085 7760000 PMC2192076

[64] Apps R Meng Z Del Prete GQ Lifson JD Zhou M Carrington M. Relative expression levels of the HLA class-I proteins in normal and HIV-infected cells. J Immunol (2015) 194(8):3594–600. 10.4049/jimmunol.1403234 25754738 PMC4390493

[65] Muczynski KA Ekle DM Coder DM Anderson SK. Normal human kidney HLA-DR-expressing renal microvascular endothelial cells: characterization, isolation, and regulation of MHC class II expression. J Am Soc Nephrol (2003) 14(5):1336–48. 10.1097/01.ASN.0000061778.08085.9F 12707403

[66] Dellgren C Nehlin JO Barington T. Cell surface expression level variation between two common Human Leukocyte Antigen alleles, HLA-A2 and HLA-B8, is dependent on the structure of the C terminal part of the alpha 2 and the alpha 3 domains. PLoS One (2015) 10(8):e0135385. 10.1371/journal.pone.0135385 26258424 PMC4530957

[67] Ramsuran V Kulkarni S O’Huigin C Yuki Y Augusto DG Gao X Epigenetic regulation of differential HLA-A allelic expression levels. Hum Mol Genet (2015) 24(15):4268–75. 10.1093/hmg/ddv158 25935001 PMC4492392

[68] Giacomini P Beretta A Nicotra MR Ciccarelli G Martayan A Cerboni C HLA-C heavy chains free of beta2-microglobulin: distribution in normal tissues and neoplastic lesions of non-lymphoid origin and interferon-gamma responsiveness. Tissue Antigens (1997) 50(6):555–66. 10.1111/j.1399-0039.1997.tb02913.x 9458108

[69] Sibilio L Martayan A Setini A Lo Monaco E Tremante E Butler RH A single bottleneck in HLA-C assembly. J Biol Chem (2008) 283(3):1267–74. 10.1074/jbc.M708068200 17956861

[70] Kulkarni S Savan R Qi Y Gao X Yuki Y Bass SE Differential microRNA regulation of HLA-C expression and its association with HIV control. Nature (2011) 472(7344):495–8. 10.1038/nature09914 21499264 PMC3084326

[71] Bryan CF Luger AM Smith JL Warady BA Wakefield M Schadde E Sharing kidneys across donor-service area boundaries with sensitized candidates can be influenced by HLA C. Clin Transplant (2010) 24(1):56–61. 10.1111/j.1399-0012.2009.01167.x 20015269

[72] Triulzi DJ Kleinman S Kakaiya RM Busch MP Norris PJ Steele WR The effect of previous pregnancy and transfusion on HLA alloimmunization in blood donors: implications for a transfusion-related acute lung injury risk reduction strategy. Transfusion (2009) 49(9):1825–35. 10.1111/j.1537-2995.2009.02206.x 19453983 PMC2841001

[73] De Clippel D Baeten M Torfs A Emonds MP Feys HB Compernolle V Screening for HLA antibodies in plateletpheresis donors with a history of transfusion or pregnancy. Transfusion (2014) 54(12):3036–42. 10.1111/trf.12727 24863861

[74] Hyun J Park KD Yoo Y Lee B Han BY Song EY Effects of different sensitization events on HLA alloimmunization in solid organ transplantation patients. Transplant Proc (2012) 44(1):222–5. 10.1016/j.transproceed.2011.12.049 22310619

[75] Salvatierra O Jr Vincenti F Amend W Potter D Iwaki Y Opelz G Deliberate donor-specific blood transfusions prior to living related renal transplantation. A new approach. Ann Surg (1980) 192(4):543–52. 10.1097/00000658-198010000-00012 6448588 PMC1347002

[76] Pfaff WW Fennell RS Howard RJ Ireland JF Scornik JC. Planned random donor blood transfusion in preparation for transplantation. Sensitization and graft survival. Transplantation (1984) 38(6):701–3. 10.1097/00007890-198412000-00030 6390835

[77] Josien R Heslan M Brouard S Soulillou JP Cuturi MC. Critical requirement for graft passenger leukocytes in allograft tolerance induced by donor blood transfusion. Blood (1998) 92(12):4539–44. 9845518

[78] Fuller TC Delmonico FL Cosimi B Huggins CE King M Russell PS Impact of blood transfusion on renal transplantation. Ann Surg (1978) 187(2):211–8. 10.1097/00000658-197802000-00020 343736 PMC1396477

[79] Scornik JC Bromberg JS Norman DJ Bhanderi M Gitlin M Petersen J. An update on the impact of pre-transplant transfusions and allosensitization on time to renal transplant and on allograft survival. BMC Nephrol (2013) 14:217. 10.1186/1471-2369-14-217 24107093 PMC4125965

[80] Regan L Braude PR Hill DP. A prospective study of the incidence, time of appearance and significance of anti-paternal lymphocytotoxic antibodies in human pregnancy. Hum Reprod (1991) 6(2):294–8. 2056027 10.1093/oxfordjournals.humrep.a137325

[81] Dunn TB Noreen H Gillingham K Maurer D Ozturk OG Pruett TL Revisiting traditional risk factors for rejection and graft loss after kidney transplantation. Am J Transplant (2011) 11(10):2132–43. 10.1111/j.1600-6143.2011.03640.x 21812918 PMC3184338

[82] Masson E Vidal C Deschamps M Bongain S Thevenin C Dupont I Incidence and risk factors of anti-HLA immunization after pregnancy. Hum Immunol (2013) 74(8):946–51. 10.1016/j.humimm.2013.04.025 23628391

[83] Higgins R Lowe D Hathaway M Williams C Lam FT Kashi H Human leukocyte antigen antibody-incompatible renal transplantation: excellent medium-term outcomes with negative cytotoxic crossmatch. Transplantation (2011) 92(8):900–6. 10.1097/TP.0b013e31822dc38d 21968524

[84] Higgins R Lowe D Daga S Hathaway M Williams C Lam FT Pregnancy-induced HLA antibodies respond more vigorously after renal transplantation than antibodies induced by prior transplantation. Hum Immunol (2015) 76(8):546–52. 10.1016/j.humimm.2015.01.012 26116896

[85] Everly MJ Rebellato LM Haisch CE Ozawa M Parker K Briley KP Incidence and impact of de novo donor-specific alloantibody in primary renal allografts. Transplantation (2013) 95(3):410–7. 10.1097/TP.0b013e31827d62e3 23380861

[86] Kaneku H O’Leary JG Banuelos N Jennings LW Susskind BM Klintmalm GB De novo donor-specific HLA antibodies decrease patient and graft survival in liver transplant recipients. Am J Transplant (2013) 13(6):1541–8. 10.1111/ajt.12212 23721554 PMC4408873

[87] Smith JD Banner NR Hamour M Ozawa M Goh A Robinson D De novo donor HLA-specific antibodies after heart transplantation are an independent predictor of poor patient survival. Am J Transplant (2011) 11(2):312–9. 10.1111/j.1600-6143.2010.03383.x 21219570

[88] Wiebe C Gibson W Blydt-Hansen TD Karpinski M Ho J Storsley LJ Evolution and clinical pathologic correlations of de novo donor-specific HLA antibody post kidney transplant. Am J Transplant (2012) 12(5):1157–67. 10.1111/j.1600-6143.2012.04013.x 22429309

[89] Billen EV Christiaans MH Lee J van den Berg-Loonen EM. Donor-directed HLA antibodies before and after transplantectomy detected by the luminex single antigen assay. Transplantation (2009) 87(4):563–9. 10.1097/TP.0b013e3181949e37 19307795

[90] Cecka JM Terasaki PI Repeating HLA antigen mismatches in renal retransplants – a second class mistake? Transplantation (1994) 57(4):515–9. 10.1097/00007890-199402000-00007 8116035

[91] Doxiadis II de Lange P D’Amaro J de Meester J Schreuder GM Claas FH Repeated HLA mismatches in cadaveric renal transplantation: is it safe to transplant? Transplant Proc (1997) 29(1–2):1408–9. 10.1016/S0041-1345(96)00612-4 9123357

[92] Fuller A Profaizer T Roberts L Fuller TC. Repeat donor HLA-DR mismatches in renal transplantation: is the increased failure rate caused by noncytotoxic HLA-DR alloantibodies? Transplantation (1999) 68(4):589–91. 10.1097/00007890-199907270-00002 10480424

[93] Farney AC Matas AJ Noreen HJ Reinsmoen N Segall M Schmidt WJ Does re-exposure to mismatched HLA antigens decrease renal re-transplant allograft survival? Clin Transplant (1996) 10(2):147–56. 8664509

[94] House AA Chang PC Luke PP Leckie SH Howson WT Ball EJ Re-exposure to mismatched HLA class I is a significant risk factor for graft loss: multivariable analysis of 259 kidney retransplants. Transplantation (2007) 84(6):722–8. 10.1097/01.tp.0000281398.41670.1f 17893605

[95] Coupel S Giral-Classe M Karam G Morcet JF Dantal J Cantarovich D Ten-year survival of second kidney transplants: impact of immunologic factors and renal function at 12 months. Kidney Int (2003) 64(2):674–80. 10.1046/j.1523-1755.2003.00104.x 12846765

[96] O’Connor MJ Lind C Tang X Gossett J Weber J Monos D Persistence of anti-human leukocyte antibodies in congenital heart disease late after surgery using allografts and whole blood. J Heart Lung Transplant (2013) 32(4):390–7. 10.1016/j.healun.2012.12.009 23395085

[97] Kwon MH Zhang JQ Schaenman JM Cadeiras M Gjertson DW Krystal CA Characterization of ventricular assist device-mediated sensitization in the bridge-to-heart-transplantation patient. J Thorac Cardiovasc Surg (2015) 149(4):1161–6. 10.1016/j.jtcvs.2015.01.003 25702320 PMC7130105

[98] Morales-Buenrostro LE Terasaki PI Marino-Vazquez LA Lee JH El-Awar N Alberu J. “Natural” human leukocyte antigen antibodies found in nonalloimmunized healthy males. Transplantation (2008) 86(8):1111–5. 10.1097/01.tp.0000331682.91699.7b 18946350

[99] Burrows SR Silins SL Khanna R Burrows JM Rischmueller M McCluskey J Cross-reactive memory T cells for Epstein-Barr virus augment the alloresponse to common human leukocyte antigens: degenerate recognition of major histocompatibility complex-bound peptide by T cells and its role in alloreactivity. Eur J Immunol (1997) 27(7):1726–36. 10.1002/eji.1830270126 9247584

[100] Amir AL D’Orsogna LJ Roelen DL van Loenen MM Hagedoorn RS de Boer R Allo-HLA reactivity of virus-specific memory T cells is common. Blood (2010) 115(15):3146–57. 10.1182/blood-2009-07-234906 20160165

[101] Morice A Charreau B Neveu B Brouard S Soulillou JP Bonneville M Cross-reactivity of herpesvirus-specific CD8 T cell lines toward allogeneic class I MHC molecules. PLoS One (2010) 5(8):e12120. 10.1371/journal.pone.0012120 20711433 PMC2920819

[102] D’Orsogna LJ van der Meer-Prins EM Zoet YM Roelen DL Doxiadis II Claas FH. Detection of allo-HLA cross-reactivity by virus-specific memory T-cell clones using single HLA-transfected K562 cells. Methods Mol Biol (2012) 882:339–49. 10.1007/978-1-61779-842-9_19 22665243

[103] de Santis C Lopalco L Robbioni P Longhi R Rappocciolo G Siccardi AG Human antibodies to immunodominant C5 region of HIV-1 gp120 cross-react with HLA class I on activated cells. AIDS Res Hum Retroviruses (1994) 10(2):157–62. 10.1089/aid.1994.10.157 7515259

[104] Alberu J Morales-Buenrostro LE de Leo C Vargas-Rojas MI Marino-Vazquez LA Crispin JC. A non-allogeneic stimulus triggers the production of de novo HLA antibodies in healthy adults. Transpl Immunol (2007) 18(2):166–71. 10.1016/j.trim.2007.06.001 18005863

[105] Ravindranath MH Kaneku H El-Awar N Morales-Buenrostro LE Terasaki PI. Antibodies to HLA-E in nonalloimmunized males: pattern of HLA-Ia reactivity of anti-HLA-E-positive sera. J Immunol (2010) 185(3):1935–48. 10.4049/jimmunol.1000424 20610644

[106] El-Awar N Terasaki PI Nguyen A Sasaki N Morales-Buenrostro LE Saji H Epitopes of HLA antibodies found in sera of normal healthy males and cord blood. Clin Transpl (2008):199–214. 10.1016/j.humimm.2009.06.020 19708457

[107] Roberts T Tumer G Gebel HM Bray RA. Solid-phase assays for the detection of alloantibody against human leukocyte antigens: panacea or Pandora? Int J Immunogenet (2014) 41(5):362–9. 10.1111/iji.12138 25066258

[108] Visentin J Guidicelli G Bachelet T Jacquelinet C Audry B Nong T Denatured class I human leukocyte antigen antibodies in sensitized kidney recipients: prevalence, relevance, and impact on organ allocation. Transplantation (2014) 98(7):738–44. 10.1097/TP.0000000000000315 25289917

[109] In JW Rho EY Shin S Park KU Song EY False-positive reactions against HLA class II molecules detected in Luminex single-antigen bead assays. Ann Lab Med (2014) 34(5):408–10. 10.3343/alm.2014.34.5.408 25187899 PMC4151015

[110] Cai J Terasaki PI Anderson N Lachmann N Schonemann C Intact HLA not beta2m-free heavy chain-specific HLA class I antibodies are predictive of graft failure. Transplantation (2009) 88(2):226–30. 10.1097/TP.0b013e3181ac6198 19623018

[111] Otten HG Verhaar MC Borst HP van Eck M van Ginkel WG Hene RJ The significance of pretransplant donor-specific antibodies reactive with intact or denatured human leucocyte antigen in kidney transplantation. Clin Exp Immunol (2013) 173(3):536–43. 10.1111/cei.12127 23627692 PMC3949641

[112] Jackson AM Sigdel TK Delville M Hsieh SC Dai H Bagnasco S Endothelial cell antibodies associated with novel targets and increased rejection. J Am Soc Nephrol (2015) 26(5):1161–71. 10.1681/ASN.2013121277 25381426 PMC4413753

[113] Sun Q Cheng Z Cheng D Chen J Ji S Wen J De novo development of circulating anti-endothelial cell antibodies rather than pre-existing antibodies is associated with post-transplant allograft rejection. Kidney Int (2011) 79(6):655–62. 10.1038/ki.2010.437 20980975

[114] Zhang Q Cecka JM Gjertson DW Ge P Rose ML Patel JK HLA and MICA: targets of antibody-mediated rejection in heart transplantation. Transplantation (2011) 91(10):1153–8. 10.1097/TP.0b013e3182157d60 21544036 PMC3563270

[115] Patel R Terasaki PI Significance of the positive crossmatch test in kidney transplantation. N Engl J Med (1969) 280(14):735–9. 10.1056/NEJM196904032801401 4886455

[116] Suitters A Rose M Higgins A Yacoub MH MHC antigen expression in sequential biopsies from cardiac transplant patients – correlation with rejection. Clin Exp Immunol (1987) 69(3):575–83. 3311497 PMC1542371

[117] Hubscher SG Adams DH Elias E. Changes in the expression of major histocompatibility complex class II antigens in liver allograft rejection. J Pathol (1990) 162(2):165–71. 10.1002/path.1711620115 2250195

[118] Facoetti A Nano R Zelini P Morbini P Benericetti E Ceroni M Human leukocyte antigen and antigen processing machinery component defects in astrocytic tumors. Clin Cancer Res (2005) 11(23):8304–11. 10.1158/1078-0432.CCR-04-2588 16322289

[119] Lefaucheur C Loupy A Hill GS Andrade J Nochy D Antoine C Preexisting donor-specific HLA antibodies predict outcome in kidney transplantation. J Am Soc Nephrol (2010) 21(8):1398–406. 10.1681/ASN.2009101065 20634297 PMC2938596

[120] Loupy A Jordan SC Transplantation: donor-specific HLA antibodies and renal allograft failure. Nat Rev Nephrol (2013) 9(3):130–1. 10.1038/nrneph.2013.18 23399581

[121] Valenzuela NM Reed EF Antibodies to HLA molecules mimic agonistic stimulation to trigger vascular cell changes and induce allograft injury. Curr Transplant Rep (2015) 2(3):222–32. 10.1007/s40472-015-0065-6 PMC5365147 28344919

[122] Zhang X Rozengurt E Reed EF HLA class I molecules partner with integrin beta4 to stimulate endothelial cell proliferation and migration. Sci Signal (2010) 3(149):ra85 10.1126/scisignal.2001158 21098729 PMC3878299

[123] Jin YP Fishbein MC Said JW Jindra PT Rajalingam R Rozengurt E Anti-HLA class I antibody-mediated activation of the PI3K/Akt signaling pathway and induction of Bcl-2 and Bcl-xL expression in endothelial cells. Hum Immunol (2004) 65(4):291–302. 10.1016/j.humimm.2004.07.014 15120184

[124] Le Bas-Bernardet S Coupel S Chauveau A Soulillou JP Charreau B. Vascular endothelial cells evade apoptosis triggered by human leukocyte antigen-DR ligation mediated by allospecific antibodies. Transplantation (2004) 78(12):1729–39. 10.1097/01.TP.0000147339.31581.99 15614145

[125] Narayanan K Jaramillo A Phelan DL Mohanakumar T. Pre-exposure to sub-saturating concentrations of HLA class I antibodies confers resistance to endothelial cells against antibody complement-mediated lysis by regulating Bad through the phosphatidylinositol 3-kinase/Akt pathway. Eur J Immunol (2004) 34(8):2303–12. 10.1002/eji.200324843 15259028

[126] Lepin EJ Zhang Q Zhang X Jindra PT Hong LS Ayele P Phosphorylated S6 ribosomal protein: a novel biomarker of ­antibody-mediated rejection in heart allografts. Am J Transplant (2006) 6(7):1560–71. 10.1111/j.1600-6143.2006.01355.x 16827856

[127] Jindra PT Hsueh A Hong L Gjertson D Shen XD Gao F Anti-MHC class I antibody activation of proliferation and survival signaling in murine cardiac allografts. J Immunol (2008) 180(4):2214–24. 10.4049/jimmunol.180.4.2357 18250428 PMC3883756

[128] Li F Wei J Valenzuela NM Lai C Zhang Q Gjertson D Phosphorylated S6 kinase and S6 ribosomal protein are diagnostic markers of ­antibody-mediated rejection in heart allografts. J Heart Lung Transplant (2015) 34(4):580–7. 10.1016/j.healun.2014.09.047 25511749 PMC4402106

[129] Ziegler ME Jin YP Young SH Rozengurt E Reed EF. HLA class I-mediated stress fiber formation requires ERK1/2 activation in the absence of an increase in intracellular Ca2+ in human aortic endothelial cells. Am J Physiol Cell Physiol (2012) 303(8):C872–82. 10.1152/ajpcell.00199.2012 22914643 PMC3469712

[130] Ziegler ME Souda P Jin YP Whitelegge JP Reed EF. Characterization of the endothelial cell cytoskeleton following HLA class I ligation. PLoS One (2012) 7(1):e29472. 10.1371/journal.pone.0029472 22247778 PMC3256144

[131] Galvani S Auge N Calise D Thiers JC Canivet C Kamar N HLA class I antibodies provoke graft arteriosclerosis in human arteries transplanted into SCID/beige mice. Am J Transplant (2009) 9(11):2607–14. 10.1111/j.1600-6143.2009.02804.x 19843036

[132] Galvani S Trayssac M Auge N Thiers JC Calise D Krell HW A key role for matrix metalloproteinases and neutral sphingomyelinase-2 in transplant vasculopathy triggered by anti-HLA antibody. Circulation (2011) 124(24):2725–34. 10.1161/CIRCULATIONAHA.111.021790 22082680

[133] Yamakuchi M Kirkiles-Smith NC Ferlito M Cameron SJ Bao C Fox-Talbot K Antibody to human leukocyte antigen triggers endothelial exocytosis. Proc Natl Acad Sci U S A (2007) 104(4):1301–6. 10.1073/pnas.0602035104 17229850 PMC1783109

[134] Valenzuela NM Hong L Shen XD Gao F Young SH Rozengurt E Blockade of p-selectin is sufficient to reduce MHC I antibody-elicited monocyte recruitment in vitro and in vivo. Am J Transplant (2013) 13(2):299–311. 10.1111/ajt.12016 23279566 PMC3563267

[135] Valenzuela NM Mulder A Reed EF HLA class I antibodies trigger increased adherence of monocytes to endothelial cells by eliciting an increase in endothelial P-selectin and, depending on subclass, by engaging FcgammaRs. J Immunol (2013) 190(12):6635–50. 10.4049/jimmunol.1201434 23690477 PMC3885237

[136] Wehner J Morrell CN Reynolds T Rodriguez ER Baldwin WM III. Antibody and complement in transplant vasculopathy. Circ Res (2007) 100(2):191–203. 10.1161/01.RES.0000255032.33661.88 17272820

[137] Nickeleit V Mihatsch MJ Kidney transplants, antibodies and rejection: is C4d a magic marker? Nephrol Dial Transplant (2003) 18(11):2232–9. 10.1093/ndt/gfg304 14551348

[138] Rodriguez ER Skojec DV Tan CD Zachary AA Kasper EK Conte JV Antibody-mediated rejection in human cardiac allografts: evaluation of immunoglobulins and complement activation products C4d and C3d as markers. Am J Transplant (2005) 5(11):2778–85. 10.1111/j.1600-6143.2005.01074.x 16212640 PMC1363343

[139] Kubagawa H Oka S Kubagawa Y Torii I Takayama E Kang DW Identity of the elusive IgM Fc receptor (FcmuR) in humans. J Exp Med (2009) 206(12):2779–93. 10.1084/jem.20091107 19858324 PMC2806608

[140] Bruhns P Iannascoli B England P Mancardi DA Fernandez N Jorieux S Specificity and affinity of human Fcgamma receptors and their polymorphic variants for human IgG subclasses. Blood (2009) 113(16):3716–25. 10.1182/blood-2008-09-179754 19018092

[141] Nimmerjahn F Ravetch JV FcgammaRs in health and disease. Curr Top Microbiol Immunol (2011) 350:105–25. 10.1007/82_2010_86 20680807

[142] Manger K Repp R Spriewald BM Rascu A Geiger A Wassmuth R Fcgamma receptor IIa polymorphism in Caucasian patients with systemic lupus erythematosus: association with clinical symptoms. Arthritis Rheum (1998) 41(7):1181–9. 10.1002/1529-0131(199807)41:7<1181::AID-ART6>3.0.CO;2-C 9663473

[143] Karassa FB Trikalinos TA Ioannidis JP. The role of FcgammaRIIA and IIIA polymorphisms in autoimmune diseases. Biomed Pharmacother (2004) 58(5):286–91. 10.1016/j.biopha.2004.04.004 15194164

[144] Diamantopoulos PT Kalotychou V Polonyfi K Sofotasiou M Anastasopoulou A Galanopoulos A Correlation of Fc-gamma RIIA polymorphisms with latent Epstein-Barr virus infection and latent membrane protein 1 expression in patients with low grade B-cell lymphomas. Leuk Lymphoma (2013) 54(9):2030–4. 10.3109/10428194.2012.762512 23270585

[145] Mellor JD Brown MP Irving HR Zalcberg JR Dobrovic A. A critical review of the role of Fc gamma receptor polymorphisms in the response to monoclonal antibodies in cancer. J Hematol Oncol (2013) 6:1. 10.1186/1756-8722-6-1 23286345 PMC3549734

[146] Maiga B Dolo A Toure O Dara V Tapily A Campino S Fc gamma receptor IIa-H131R polymorphism and malaria susceptibility in sympatric ethnic groups, Fulani and Dogon of Mali. Scand J Immunol (2014) 79(1):43–50. 10.1111/sji.12122 24117665 PMC3992902

[147] Pawlik A Florczak M Bak L Domanski L Rozanski J Dabrowska-Zamojcin E The Fc gamma RIIa polymorphism in patients with acute kidney graft rejection. Ann Transplant (2003) 8(4):24–6. 15171001

[148] Yuan FF Watson N Sullivan JS Biffin S Moses J Geczy AF Association of Fc gamma receptor IIA polymorphisms with acute renal-allograft rejection. Transplantation (2004) 78(5):766–9. 10.1097/01.TP.0000132560.77496.CB 15371685

[149] Berry GJ Burke MM Andersen C Bruneval P Fedrigo M Fishbein MC The 2013 International Society for Heart and Lung Transplantation Working Formulation for the standardization of nomenclature in the pathologic diagnosis of antibody-mediated rejection in heart transplantation. J Heart Lung Transplant (2013) 32(12):1147–62. 10.1016/j.healun.2012.11.005 24263017

[150] Fishbein GA Fishbein MC. Morphologic and immunohistochemical findings in antibody-mediated rejection of the cardiac allograft. Hum Immunol (2012) 73(12):1213–7. 10.1016/j.humimm.2012.07.011 22813651

[151] Fedrigo M Feltrin G Poli F Frigo AC Benazzi E Gambino A Intravascular macrophages in cardiac allograft biopsies for diagnosis of early and late antibody-mediated rejection. J Heart Lung Transplant (2013) 32(4):404–9. 10.1016/j.healun.2012.12.017 23498161

[152] Xu L Collins J Drachenberg C Kukuruga D Burke A. Increased macrophage density of cardiac allograft biopsies is associated with antibody-mediated rejection and alloantibodies to HLA antigens. Clin Transplant (2014) 28(5):554–60. 10.1111/ctr.12348 24580037

[153] Magil AB Tinckam K. Monocytes and peritubular capillary C4d deposition in acute renal allograft rejection. Kidney Int (2003) 63(5):1888–93. 10.1046/j.1523-1755.2003.00921.x 12675868

[154] Tinckam KJ Djurdjev O Magil AB. Glomerular monocytes predict worse outcomes after acute renal allograft rejection independent of C4d status. Kidney Int (2005) 68(4):1866–74. 10.1111/j.1523-1755.2005.00606.x 16164665

